# The Heel of Achilles in Hospital Diet

**Published:** 1913-11-22

**Authors:** 


### THE SERVICE OF PATIENTS' FOOD.

### The Heel of Achilles in Hospital Diet.

In the series of articles which has appeared in The Hospital from time to time expressing the Patients' Point of View the food given in hospitals has met with a good deal of criticism. Since, however, good food is often rendered indifferent, and indifferent food rendered uneatable, by faulty cooking or service, this, the real heel of Achilles in hospital diet, deserves close attention.

One of the morals, then, which these articles have driven home is that, while the food in hospitals is usually very good, the service of the food is often atrocious.

With regard to cooking, there should not be the slightest difficulty in deciding whether the cook is doing her work well or otherwise, for, the proof of the pudding being in the eating, her efforts are put to a constant and severe test. Of more practical importance is the custom in some hospitals of allowing the chief cook to prepare meals for the staff and the assistant cook to look after the patients' food. Such an arrangement is obviously not a good one, since the principal members of the staff may consider the cooking quite satisfactory and not realise that the food sent to the wards may be very different. In any case the direct responsibility of the chief cook for the patients' food should be insisted on, and no relegation of duty allowed to excuse food badly cooked or unappetising in appearance.

Presuming the food to have passed the cooking stage, the first difficulty is to ensure that it reaches the patients as hot, generally speaking, as it leaves the ovens. Into this many factors enter. The carving, of course, must be done on a hot-plate, and the diets (so far, at least, as the meat is concerned) placed straightway into heated food-tins. The latter may be of several types, being heated either by steam or by hot water, and containing two trays or a dozen. Whatever the type, constant watch must be kept upon them to see that they are not in need of repair, as a slight leakage in any way will quickly lower the temperature. Special attention should be paid to the fastening of the doors. If it is not possible for the tins to be taken away as soon as they are filled, their inner jackets should be so constructed that they may be connected directly with the steam or hot-water system to ensure their keeping at the same degree of heat. Even though the heat be retained, the portions of food will not keep their freshness if left standing too long, so that the whole process of serving up, carving, weighing, and delivering should be done in the very shortest time possible, every available member of the kitchen staff, as well as one or two porters, being utilised. For the actual delivery to the wards from the central kitchen a small service lift is almost essential, as the tins may be sent up on this one by one and collected on the different floors by the wardmaids. Where such a lift does not exist, a trolley must be used, ascending by means of the passenger lift. Such a procedure is not only cumbersome and inconvenient (especially where there is only one lift), but it is very slow, and half an hour may easily elapse between the times at which the first and last wards respectively are served.

Having reached the ward, the care of the food will be mainly in the hands of the sister. The portions must, of course, be taken expeditiously from the tins, which, being now open, will tend to cool rapidly. Ample assistance should be at hand to add the vegetables and to distribute the diets round the ward. Special attention should be paid to such condiments as salt, the absence of which will mean a great deal to the patient, though in the general bustle he may not get an opportunity of asking for it, or may not possess sufficient assurance to do so. The same remark applies to sugar in the case of stewed fruit or pies. The chief responsibility here will no doubt rest with the kitchen, but as sufficient sugar is rarely put in in the cooking some must be added in the serving, and if this is neglected the "afters" may be quite spoilt. Moreover, in this case, more than in that of salt, the patient will hesitate to give trouble by asking for this extra luxury. To avoid such a position, the patients should be invited to utter freely reasonable demands of this kind.

What has been said so far applies almost entirely to the dinner. The other meals, which are in a sense subsidiary, will usually be prepared in the ward kitchens, but the same care and the same supervision of detail are equally necessary and are very often neglected. Tea, for instance, is a beverage much appreciated by nearly everyone, and yet in how few cases in hospital is tea properly made! The fault does not always lie in the tea itself, though sometimes false economy leads to the purchase of leaves that yield practically none of the refreshment which is their sole purpose. The cause of the failure to make tea properly is generally pure carelessness, since the process is not a difficult one. Such carelessness, however, implies a corresponding lack of supervision. Bread-and-butter furnishes another illustration of good materials so often badly used. Rough, mis-shapen slices of bread only partly covered with butter are very uninviting and very common, and yet with the same bread and the same butter a quite appetising meal could be prepared were the supervision of the service not neglected.

### Crockery and Ward Funds.

Turning to the question of the food utensils, a large field for intelligent effort and kindly consideration is opened up. It is, unfortunately, by no means uncommon to give patients mugs for tea, without saucers and perhaps without handles. It is true that such a practice may diminish the breakages, and that some of the patients may have the same things at home; these arguments cannot justify the cold and pauper-like appearance of such utensils. The general question of crockery is, no doubt, a difficult one, but it is sometimes overcome in an admirable manner. Thus where patients give donations for the special benefit of the ward they have been in (a common practice), the amount is paid into the general funds of the institution but is credited to the ward in an account book, so that the sister is entitled to spend up to that amount in articles for the ward. Such articles will usually take the form of cups and saucers and similar small items for the comfort of the patients. While it is to the interest of the sister not to spend too much in depletion of her balance, she is yet allowed some scope for friendly rivalry with other wards, and the patients derive the benefit of little extras of which a rigid economy might otherwise deprive them.

### A Task for Visitors.

On the general question of the responsibility for the service of food mention has already been made of the cook and the ward sister as primarily responsible. Above them are, of course, the steward or housekeeper on the one hand, and the matron on the other. These latter officers have a great share of responsibility, and it should be their duty to keep in very close touch, both by sight and by taste, with the food as served to individual patients. Beyond them come the governors of the institution and, more particularly, the house committee and the monthly visitors. These persons should endeavour to make themselves thoroughly acquainted with the question of the patients' food, and should go round the wards at meal-times with the special object of tasting it. To do this requires perhaps more courage than appears at first sight, more especially when the visitor is accompanied round the ward by a charming and conversational sister. However, this duty is one that cannot be shirked if the visitor is to be of real use. Another officer who has a direct responsibility in the matter of the food is the visiting physician or surgeon, who, however, rarely shows any interest.

This is the more strange, since the doctor's word is law in such a connection, and it is very easy for a patient (or a whole group of patients) to be fed day after day, week after week, and month after month on (for instance) mutton and rice pudding, mutton and rice pudding, without the slightest variation, simply because a member of the medical staff has at some time given directions to that effect and his successors have not troubled to ascertain what really goes on.

It is obvious, therefore, that the entire responsibility for any errors that may occur in the service of food cannot all be laid at the door of any one person, and that only by the zealous co-operation of all the officers who have been mentioned can the service be rescued from its present neglect and inefficiency.

Finally, it must not be overlooked that conditions are changing in respect of the class of patient admitted to the voluntary hospitals, and that the encouragement of (or demand for) payment from patients is increasing. Such are admitted facts, and they cannot but have an effect upon the question of the service of food, since the hospitals which continue to countenance neglect in this respect will soon learn from pay-patients that good food with bad service is good food wasted. For patients accustomed to appetising meals it would be very unfortunate if, in coming to hospital for skilled nursing and the highest medical knowledge, they should be expected to forego such things as these, which, while meaning a great deal to the recipient, cost little in point of money, but demand the attention and supervision which is now so grudgingly given as to make the service of food the Cinderella of hospital departments.

